# Immediate clinical outcomes of left bundle branch area pacing vs conventional right ventricular pacing

**DOI:** 10.1002/clc.23215

**Published:** 2019-06-11

**Authors:** JunMeng Zhang, Zefeng Wang, Liting Cheng, Linna Zu, Zhuo Liang, Fei Hang, Xinlu Wang, Xiaoyan Li, Ruijuan Su, Jie Du, Yongquan Wu

**Affiliations:** ^1^ Department of Cardiology, Beijing Anzhen Hospital Capital Medical University Beijing China; ^2^ Beijing Anzhen Hospital Capital Medical University Beijing China; ^3^ The Key Laboratory of Remodeling‐Related Cardiovascular Diseases, Capital Medical University, Ministry of Education, Beijing Institute of Heart Lung and Blood Vessel Diseases Beijing China; ^4^ Department of Ultrasound Beijing Jishuitan Hospital Beijing China

**Keywords:** left bundle branch area pacing, physiological pacing, QRS complex, right ventricular pacing

## Abstract

**Background:**

Left bundle branch area pacing (LBBaP) is a new physiological pacing strategy that produces comparable clinical effects to His bundle pacing (HBP).

**Objective:**

The purpose of this study was to investigate the immediate clinical outcomes of LBBaP vs RVP.

**Methods and Results:**

From April 2018 to September 2018, we included 44 patients under continuous pacemaker implantation. Patients were randomly divided into the LBBaP group and conventional RVP group. Compared to the RVP group, the LBBaP group displayed significantly increased operative (90.10 ± 19.68 minutes vs 61.57 ± 6.62 minutes, *P* < .001) and X‐ray exposure times (15.55 ± 5.62 minutes vs 4.67 ± 2.06 minutes, *P* < .001). The lead threshold of the LBBaP group was increased (0.68 ± 0.20 mV vs 0.51 ± 0.0 mV, *P* = .001), while the R‐wave amplitude and ventricular impedance did not significantly differ between the two groups. The conventional RVP procedure significantly widened the QRS complex (93.62 ± 8.28 ms vs 135.19 ± 12.21 ms, *P* = .001), whereas the LBBaP had no effect on QRS complex (130.13 ± 43.30 ms vs 112.63 ± 12.14 ms, *P* = .904). Furthermore, the LBBaP procedure significantly narrowed the QRS complex in patients with left bundle branch block (LBBB) (168.43 ± 38.870 ms vs 119.86 ± 6.69 ms, *P* = .019).

**Conclusion:**

LBBaP is a new physiological, safe and effective pacing procedure with a high overall success rate. Compared to conventional RVP, LBBaP can correct LBBB, thereby improving cardiac electrical dyssynchrony.

**ABBREVIATIONS**
AF: atrial fibrillation
CRT: cardiac resynchronization therapy
ECG: electrocardiogram
HBP: His bundle pacing
HF: heart failure
HFH: heart failure hospitalization
HYHA: New York Heart Association
LAO: left anterior oblique
LBB: left bundle branch
LBBaP: left bundle branch area pacing
LV: left ventricular
LVDD: left ventricular end‐diastolic dimension
LVEF: left ventricular ejection fraction
RAO: right anterior oblique
RBB: right bundle branch
RV: right ventricular
RVP: right ventricular pacing

## 1 INTRODUCTION

Right ventricular pacing (RVP) is gradually being recognized as a conventional pacing method which may lead to cardiac electro‐mechanical dyssynchronize.^1^ Most recent studies reveal that RVP increases the morbidity of atrial fibrillation (AF) and heart failure (HF), also RVP increase the hospitalization rate of heart failure and mortality in the patients with high pacing ratio.^2^, ^3^, ^4^

Although clinical experts have attempted pacing multiple positions (apical, interval, right ventricular outflow tract) in the right ventricle, these do not lead to physiological pumping of the heart and so the clinical effect does not make a difference.^5^ Deshmukh et al^6^ first reported His bundle pacing (HBP) as a safe and effective physiological pacing method in patients with chronic AF. Since then, a number of clinical studies have demonstrated its feasibility and effectiveness, the indications of which are expanding.^7^, ^8^, ^9^, ^10^, ^11^ Consequently, HBP is currently recognized as a major physiological pacing method. However, several shortcomings of HBP have been identified in clinical practice, including a high pacing threshold, lead dislocation rate, and low success rate among pacemaker implantation methods, particularly in patients with conduction block at sites distant from His bundle.^9^, ^12^, ^13^ Huang et al^14^ first created the Left bundle branch area pacing (LBBaP) procedure. A total of 3830 pacing lead was positioned in standard his pacing location, noting His potential, the lead was then advanced into the interventricular septum to reach the left bundle branch area (LBBa), in which allowing a lower output to correct LBBB to achieve physiological pacing. The lead parameters using this procedure after 1 year of follow‐up remained favorable. Medtornic inc. 3830 lead when used in introduction, this is done later in the paper, but should be used throughout to identify pacing lead being used. In this context, this study detailed the operation procedure and criteria of LBBaP and also provided a comparison of this procedure to RVP.

## 2 METHODS

### 2.1 Study population

All consecutive patients from Beijing Anzhen Hospital received implantable pacemakers between July and September 2018, according to the established guidelines.^15^ The patients in this study were split up into two groups based on the pacing site of the right ventricular (RV); one group of patients received traditional right ventricular apex pacing (RVP group) and the other group received LBBaP area pacing (LBBaP group). Our study was performed according to the ethical guidelines of the 1975 Declaration of Helsinki and approved by the Research Ethics Committee of Capital Medical University affiliated to Beijing Anzhen Hospital. All patients provided written informed consent.

## 3 IMPLANTATION PROCEDURE

### 3.1 LBBaP implantation

**Figure 1:**
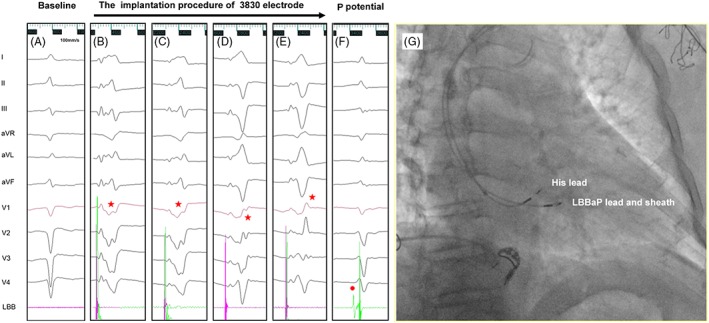
The procedure of left bundle branch (LBB) area implantation with the 3830 lead. A, The Baseline ECG of the patient with left bundle branch area pacing (LBBaP); B to E represent the process of 3830 lead implantation: With 2 V output and unipolar paced lead, the V1 QRS wave appeared W‐shaped with a mid‐notch (B); The leads were intermittently paced and the V1 QRS morphology (notch) gradually moved back until the vertical R wave appeared in the form of right bundle branch block (the red pentagram shows); F, the 3830 lead implanted in LBB area successfully and P potential (the red dot shows) can be seen in the intraluminal electrogram. G, The direction of LBBaP lead and sheath to move in fluoroscopic imaging

**Figure 2:**
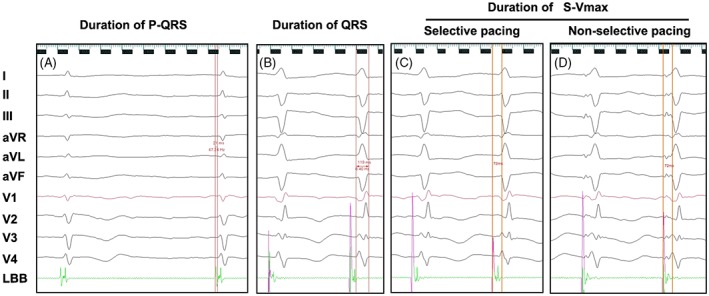
The basic criteria of left bundle branch area pacing (LBBaP). A, P potential can be seen in intracardiac electrogram and the duration of P‐QRS was 21 ms (*P* potential to the onset of QRS); B. The duration of QRS <120 ms (119 ms); C and D. The morphology (the left anterior branch block) and duration of the stimulus to the ventricular activation peak (S‐Vmax) was similar between selective and non‐selective pacing

The LBBap lead was initially placed into typical his‐bundle pacing region, which was performed as described for the HBP method.^11^, ^12^, ^14^ Briefly, with the aid of the C315 sheath (Medtronic Inc., Minneapolis, Minnesota), the selected Secure lead (model 3830, Medtronic, Inc.) was inserted into the His bundle, and then His potential was mapped and recorded first in right anterior oblique (RAO) 30°. Subsequently, the 3830 lead and C315 sheath were pushed together clockwise in the ventricular apex direction (1‐3 cm)(Figure 1G). When the 2 V output was unipolar paced by the tip of 3830 lead, the V1 QRS wave appeared W‐shaped, which was used as the ideal lead insertion point (Figure 1B); Then, under the left anterior oblique position (LAO) 30° to 45°, the C315 sheath was adjusted in the vertical direction of the RV septum (Figure 1H), which needed to rotate the sheath counterclockwise mostly; Next, the lead was screwed in with 8 to 10 clockwise rotation. The leads were intermittently paced and the V1 QRS morphology (the W‐shaped “notch”) moved back gradually until the vertical R wave appeared in the form of right bundle branch block (RBBB) and the duration of QRS narrowed (Figure 1C‐E). The threshold test were executed during the procedure and the intracardiac signals showed the Purkinje (P) potential in most cases. The unipolar pacing was narrowed by QRS, showing a left anterior branch block pattern, which signified the successful implantation of the lead (Figure 2B).

The basic criteria of LBBaP (Figure 2A‐D) were as follows: (a) The duration of QRS <120 ms; (b) P potential to QRS (P‐QRS) < his‐QRS (H‐QRS); (c) the isoelectric line from stimulus to QRS onset was identified when pacing with a low output; (d) the duration of the stimulus to the ventricular activation peak (S‐V_max_) was similar for selective and non‐selective pacing; (e) the paced morphology was the left anterior branch block; and (f) under the X‐ray, the final position of the leads in the patient performed HBP and LBBaP at the same time (**Appendices S1 and S2**).

### 3.2 RV pacing

RV leads were implanted in a standard fashion at the RV apex or septum.

### 3.3 Statistics analyses

SPSS version 19.0 (IBM, Armonk, New York) was used for all statistical analyses. Normally distributed continuous data were expressed as the mean ± SD. Categorical data were described as the number (%) and χ^2^ test or Fisher's exact test (if the sample size was less than 40 or the minimum theoretical frequency was less than 1) and used to examine the aforementioned differences. All the tests were two‐sided. A *P*‐value < .05 was considered statistically significant.

## 4 RESULTS

### 4.1 Study group

**Figure 3:**
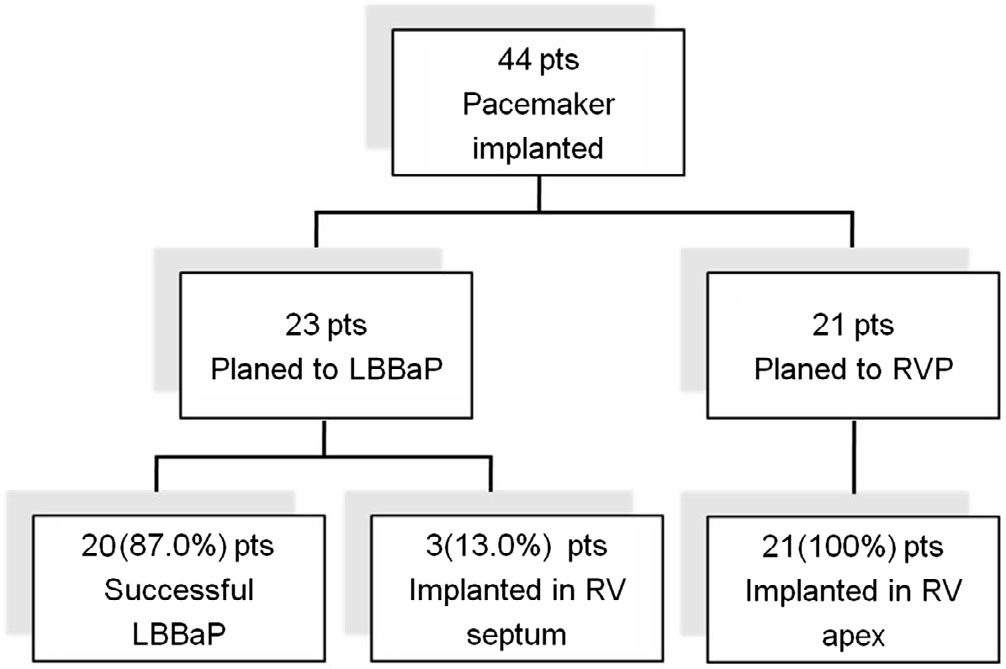
Schematic diagram of the study.;LBBaP, left bundle branch area pacing; Pts, patients; RV, right ventricular; RVP, right ventricular pacing

A total of 44 consecutive patients were enrolled and divided into two groups by the methods of RV lead implantation, that is, RVP and LBBaP groups. In the LBBaP group, 23 patients underwent pacing in the LBBa region, among which the surgery was successful in 20 patients (87.0%). A total of three patients (13.0%) failed LBBaP and underwent RV septum pacing instead. In the RVP group, 21 patients underwent conventional RVP and all surgeries were success (100%) (Figure 3). The mean age of the patients was 65.2 ± 12.9 years and included 27 males (61.4%). The basic clinical details of the patients are shown in Table 1. Compared to the RVP group, the LBBaP group displayed higher non‐ischemic cardiomyopathy (26.1% vs 0%, *P* = .012). In the LBBaP group, a higher incidence of heart failure was observed (39.1% vs 0%, *P* = .001); left ventricular ejection fraction (LVEF) was lower (45.75 ± 18.47% vs 65.93 ± 4.16%, *P* = .001), left ventricular end‐diastolic dimension (LVDD) was larger (59.79 ± 14.29 mm vs 65.93 ± 4.16 mm, *P* = .008), and the electrocardiogram(ECG) QRS interval was wider (131.83 ± 41.68 ms vs 93.62 ± 8.28 ms, *P* < .001). Other clinical baseline data did not statistically differ between the two groups.

**Table 1:** Baseline clinical and demographic characteristics of patients

	LBBaP group N = 23	RVP group N = 21	*P*‐value
Age, mean (SD)	64.61 ± 12.65	65.76 ± 13.53	.772
Male, N (%)	17 (73.9%)	10 (47.6%)	.074
Hypertension, N (%)	11 (47.8%)	9 (42.9%)	.741
Diabetes, N (%)	6 (26.1%)	1 (4.8%)	.053
Coronary artery disease, N (%)	4 (17.4%)	6 (28.6%)	.377
Ischemic stroke, N (%)	1 (4.3%)	0 (0%)	.338
Ischemic cardiomyopathy, N (%)	2 (8.7%)	0 (0%)	.167
Non‐ischemic cardiomyopathy, N (%)	6 (26.1%)	0 (0%)	.012
Atrial fibrillation, N (%)	5 (21.7%)	2 (9.5%)	.269
Heart failure, N (%)	9 (39.1%)	0 (0%)	.001
NYHA class II N (%)	5 (21.7%)		
NYHA class III N (%)	3 (13%)		
NYHA class IV N (%)	1 (4.3%)		
Sinus node dysfunction, N (%)	11 (47.8%)	15 (71.4%)	.112
AV conduction disease, N (%)	8 (38.4%)	6 (28.6%)	.659
UCG			
LVEF%, mean (SD)	45.75 ± 18.47	65.93 ± 4.16	<.001
LVDD (mm), mean (SD)	59.79 ± 14.29	47.43 ± 3.18	.008
QRS duration (ms), mean (SD)	131.83 ± 41.68	93.62 ± 8.28	<.001

AV, atrioventricular; LBBaP, left bundle branch area pacing, RVP, right ventricular pacing, NYHA , New York Heart Association Class, UCG, ultrasonic cardiogram, LVDD, left ventricular end diastolic dimension; LVEF, left ventricular ejection fraction.

Values are mean (SD), or number (%). *P*‐value <.05 was considered statistically significant.

### 4.2 Implantation results

Among the 20 patients who successfully underwent LBBaP surgery, 19 (95%) were implanted with dual‐chamber pacemaker and 1 (5%) was implanted with single‐chamber pacemaker. On the other hand, 21 patients of the RVP group were implanted with a dual‐chamber pacemaker. Compared to the RVP group, the surgery time (90.10 ± 19.68 minutes vs 61.57 ± 6.62 minutes, *P* < .001) and X‐ray exposure time (15.55 ± 5.62 minutes vs 4.67 ± 2.06 minutes, *P* < .001) were significantly increased for the LBBaP group. Intraoperative testing of the ventricular lead parameters showed that the LBBaP group lead captured the threshold increase (0.68 ± 0.20 mV vs 0.51 ± 0.06 mV, *P* = .001), while the R wave amplitude did not significantly differ between the two groups. In addition, no surgery‐related complications occurred in either group (Table 2).

**Table 2:** Procedural and immediate clinical outcomes in patients

	LBBaP group N = 20	RVP group N = 21	*P*‐value
Procedure duration (min), mean (SD)	90.10 ± 19.68	61.57 ± 6.62	<.001
Fluoroscopy duration (min), mean (SD)	15.55 ± 5.62	4.67 ± 2.06	<.001
Capture threshold (V), mean (SD)	0.68 ± 0.20	0.51 ± 0.06	.001
R wave amplitude (mV), mean (SD)	9.28 ± 5.00	11.03 ± 3.14	.185
Ventricular impedance (Ohms), mean (SD)	846.80 ± 198.45	949.71 ± 479.34	.394
Dual chamber PPM (DDD), N (%)	19 (95%)	21 (100%)	.300
Single chamber PPM (VVI), N (%)	1 (5.0%)	0 (0%)	.300
Complication, N (%)	0 (0%)	0 (0%)	—

LBBaP, left bundle branch area pacing, RVP, right ventricular pacing.

Values are mean (SD), or number (%). *P*‐value <.05 was considered statistically significant.

### 4.3 ECG characteristics

We compared the electrocardiogram (ECG) parameters before and after surgery. In the LBBaP group (n = 20), the QRS wave narrowed (130.13 ± 43.30 ms vs 112.63 ± 12.14 ms, *P* = .094). Furthermore, we analyzed patients with LBBB in the LBBaP group (n = 7) and observed significantly narrowed QRS waves (168.43 ± 38.87 ms vs 119.86 ± 6.69 ms, *P* = .019). The traditional RV pacing procedure (n = 21) significantly widened the QRS complex (93.62 ± 8.28 ms vs 135.19 ± 12.21 ms, *P* = .001). (Table 3 and Appendix S1).

**Table 3:** QRS duration before and after Pacemaker implantation. (ms), mean (SD)

	Before	After	*P*‐value
LBBaP group (N = 20)	130.13 ± 43.30	112.63 ± 12.14	.094
Patients with LBBB			
LBB‐area pacing (N = 7)	168.43 ± 38.87	119.86 ± 6.69	.019
RVP group (N = 21)	93.62 ± 8.28	135.19 ± 12.21	<.001

LBBaP, left bundle branch area pacing, LBBB, left bundle branch block, RVP, right ventricular pacing.

Values are mean (SD = SD), *P*‐value <0.05 was considered statistically significant.

## 5 DISCUSSION

This study described a new physiological pacing procedure known as LBBaP, first reported by Huang et al.^14^ In this study, we analyzed the safety and efficacy of LBBaP in a larger group of patients (n = 23) and compared it to traditional RV pacing.

The main findings were that (a) LBBaP is a safe and effective physiological pacing procedure with a high success rate (87.0%), and (b) LBBaP does not increase the QRS complex width, particularly in patients with LBBB, and significantly narrows the QRS interval.

The conventional RVP method results in artificial LBBB‐like ventricular activation and lost ventricular electromechanical synchronization. This significantly increases the incidence of AF, hospitalization rates, and mortality due to HF.^2^, ^3^, ^4^ Deshmukh et al first demonstrated the safety and efficacy of HBP as a physiological pacing method in patients with chronic AF.^6^ Subsequently, a large number of clinical studies on HBP were performed and both continuous improvement and increased indications were reported. These included its lack of use in patients with normal heart functions, such as SSS, AVB, and its capacity to change the prognosis of patients with severe HF and LBBB, achieving the effect of cardiac resynchronization therapy (CRT). Although HBP represents an important method of physiological pacing, it is associated with certain shortcomings including a high lead threshold, lead dislocation rate, and low success rate, particularly in those with His conduction block.^12^

Huang et al^14^ were the first to propose the LBBaP procedure. After the failure of HBP for high capture threshold, a 3830 lead was successfully placed onto the LBB area. The pacing output was 0.5 vs @ 0.5 ms and the procedure could correct the LBBB with accompanying right BBB on ECGs. Approximately 1 year later, the patient had a well clinical outcome and favorable echocardiographic parameters. Meta‐analysis^9^ revealed an average pacing threshold of 1.71 V for new HBP implantation. However, in this study, the threshold of the LBBaP group was higher than that of the RVP group (0.68 ± 0.20 V vs 0.51 ± 0.06 V, *P* = .001), the average threshold of 0.68 V and the pulse width of 0.5 ms were lower than the output threshold of HBP. The R wave amplitude of the lead in 20 patients with LBBaP was 9.28 ± 5.00 mV which did not statistically differ from the RVP group. The R wave amplitude of HBP was significantly increased compared to previous reports. Abdelrahman et al^4^ demonstrated that the R wave amplitude was 4.93 ± 3.46 mV in 304 patients with HBP.

In this study, the LBBaP procedure reduced the QRS interval (130.13 ± 43.30 ms vs 112.63 ± 12.14 ms, *P* = .094). Surprisingly, LBBaP could significantly reduce the QRS interval (168.43 ± 38.87 ms vs 119.86 ± 6.69 ms, *P* = .019) in patients with LBBB. The LBBaP procedure rotated the 3830 lead into the interventricular septum and allowed it to reach the left ventricular surface LBB area, directly pacing the conduction system and rapidly agitating the entire ventricle to achieve electro‐mechanical synchronization. A total of seven patients with HF combined with LBBB through the LBBaP had achieved electrical synchronization. Hence, LBBaP could significantly improve the clinical prognosis of patients with cardiac asynchrony, and achieve CRT efficacy, which may represent an alternative strategy to traditional CRT. The LBBaP procedure was similar to HBP since the heart was electrically synchronized and would be effective for patients with distant sites at His. At the same time, the advantages of a low threshold, high sensing, and firm lead were achieved. In addition, LBBaP can theoretically achieve cardiac resynchronization in patients blocked in His bundle.^14^

In this study, the surgery failed in three patients with LBBaP, accounting for 13.0% of the total patients. The reasons for the failure were as follows: When two of the patients underwent removal of the C315 sheath, the 3830 lead was pulled which dislocated the lead. Fortunately, the parameters for right ventricular septal pacing were favorable. In another case, there was an anterior myocardial infarction with severe fibrosis and the 3830 lead could not be screwed at multiple locations.

Compared to the conventional RVP procedure, the LBBaP operation and X‐ray exposure times were extended, which could be attributed to the fact that LBBaP is a new type of surgery and as such requires a learning process. During the later period, the LBBaP operation time and X‐ray exposure time were significantly shortened.

For utilizing the LBBaP procedure more widely in the future, we recommend the following instructions: (a) The 3830 lead better should be placed under the X‐ray RAO and the lead under LAO should be screwed to ensure that the pre‐shaped sheath is perpendicular to the interventricular septum surface; (b) during the screw‐in process, the intermittent lead parameter should be measured and when the impedance drops by approximately 40% of the initial value, which highly suspected that the 3830 lead entered the LV through the interventricular septum; (c) upon entering the LV through the interventricular septum, calm should be retained for favorable results when the lead is retracted; (d) for patients with an enlarged heart, to quickly identify the ideal screw‐in point, the C315 sheath should be properly shaped. After the inner sheath and outer sheath are assembled, the large bending angle of the sheath will be gently reduced; (e) patients with LBBB must have temporary pacemaker protection. Upon searching for the ideal screw point of the C315 sheath, it would be easy to damage the vulnerable RBB, so the patient will have a third‐degree atrioventricular block; and (f) for patients with myocardial infarction, particularly in patients with anterior myocardial infarction, a clear magnetic resonance imaging should be performed prior to surgery to clarify whether the interventricular septum is fibrotic. If fibrosis is evident in the interventricular septum, then the patients are not suitable for LBBaP.

## 6 STUDY LIMITATIONS

There were several limitations associated with our study. First, it was not a random, controlled, multicenter prospective study, therefore, the LBBaP surgery was not representative. Second, the small sample size may produce bias and not all patients received a follow‐up. As such, the clinical effect of LBBaP remains unexplained.

## 7 CONCLUSION

In summary, LBBaP is a new type of safe and effective physiological pacing, with an overall high success rate. With increased surgical proficiency and continuous improvement, success rates might further improve. Similar to HBP, LBBaP can achieve cardiac electrical synchronization. Compared to RVP, LBBaP does not increase patient electrical disconnection. In addition, LBBaP can correct LBBB, thereby improving electrical synchronization.

## CONFLICT OF INTEREST

The authors declare that there are no conflicts of interest regarding the publication of this paper.

## Supporting information

APPENDIX S1The morphology and duration of QRS in different pacing modes. A, Normal QRS morphology in the patient without conduction disease;
B, LBBB pattern and wide QRS interval in the RVP patient with a complete atrioventricular block.
**C** and D, LBBB pattern and the wide QRS interval before LBBaP, while left anterior branch block pattern and narrow QRS interval after LBBaP in the same patient with LBBB.
LBBB, left bundle branch block; LBBaP, left bundle branch area pacing

**APPENDIX S2** The final position of leads in one patient with HBP and LBBaP at the same time. HBP, His bundle pacing; LBBaP, left bundle branch area pacing
