# Supplementary figures and images for: Immediate clinical outcomes of left bundle branch area pacing vs conventional right ventricular pacing
Source: Clin Cardiol. 2019 Jun 11;42(8):768–73. doi: 10.1002/clc.23215 (PMC6671779; doi:10.1002/clc.23215)

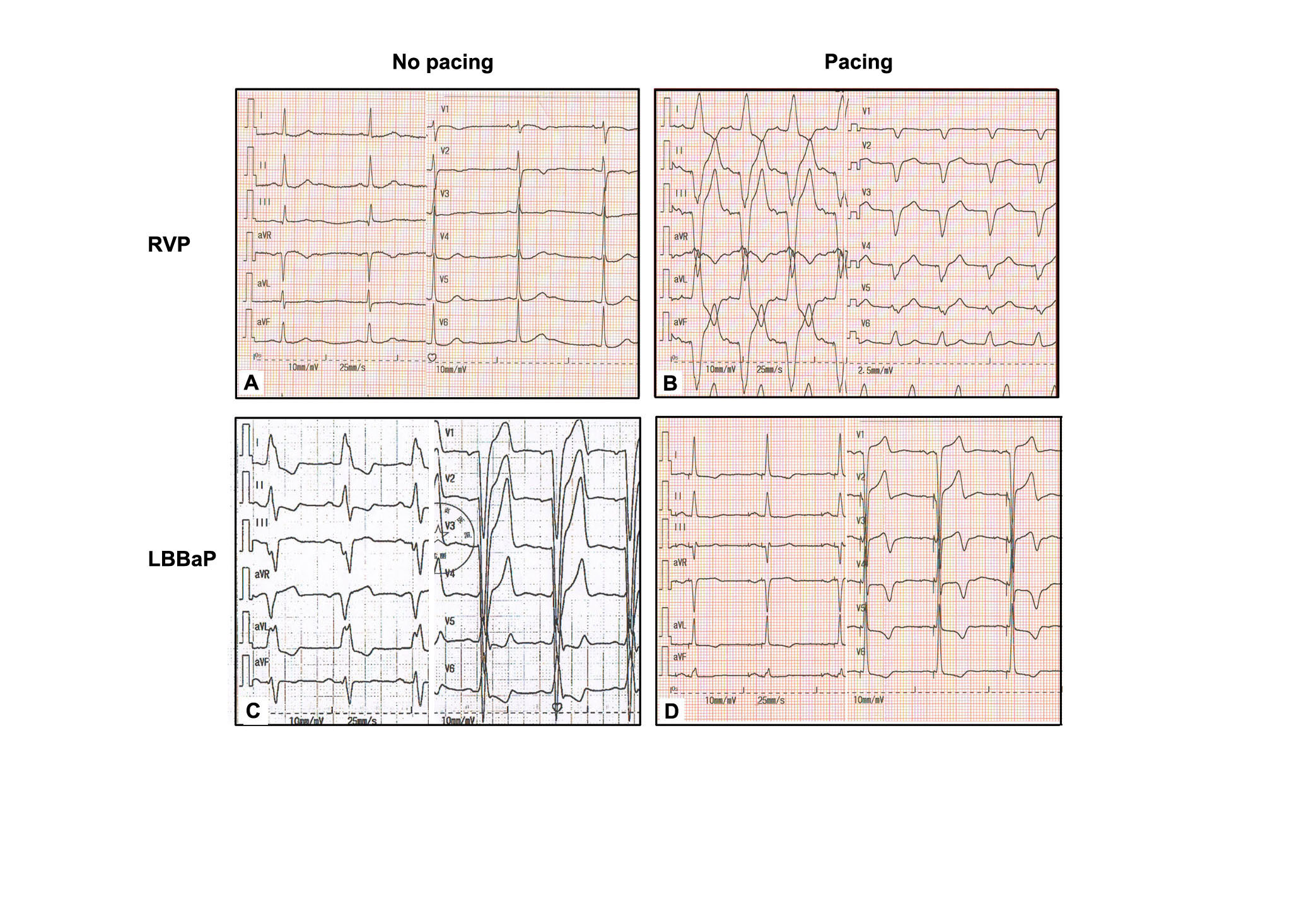

Supplement: Supplementary file 1 — APPENDIX S1The morphology and duration of QRS in different pacing modes. A, Normal QRS morphology in the patient without conduction disease; B, LBBB pattern and wide QRS interval in the RVP patient with a complete atrioventricular block. C and D, LBBB pattern and the wide QRS interval before LBBaP, while left anterior branch block pattern and narrow QRS interval after LBBaP in the same patient with LBBB. LBBB, left bundle branch block; LBBaP, left bundle branch area pacing [file CLC-42-768-s001.jpg]

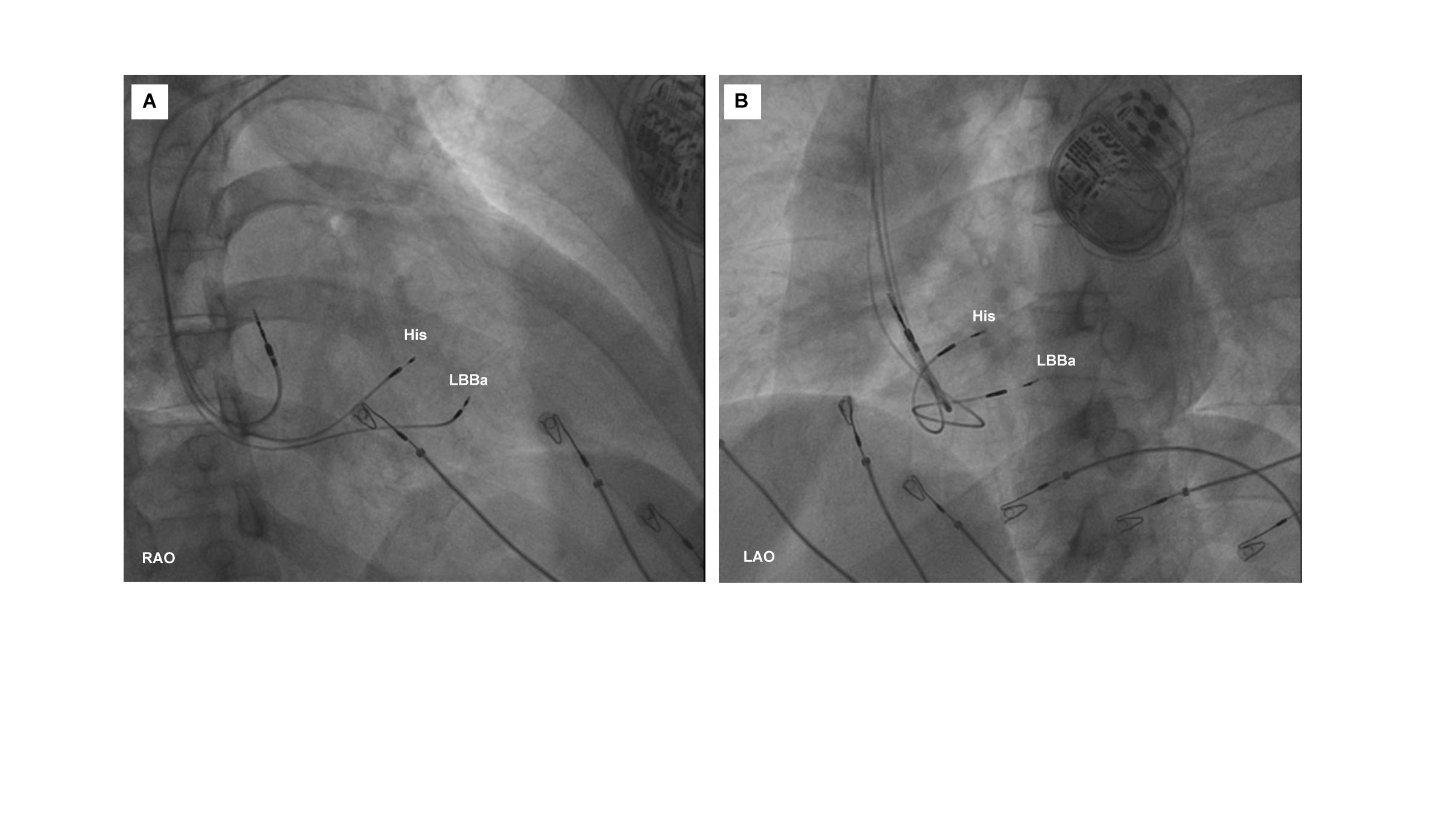

Supplement: Supplementary file 2 — APPENDIX S2 The final position of leads in one patient with HBP and LBBaP at the same time. HBP, His bundle pacing; LBBaP, left bundle branch area pacing [file CLC-42-768-s002.jpg]
